# The Role of Emotion in Global Warming Policy Support and Opposition

**DOI:** 10.1111/risa.12140

**Published:** 2013-11-12

**Authors:** Nicholas Smith, Anthony Leiserowitz

**Affiliations:** 1 Division of Psychology and Language Sciences, University College LondonLondon, WC1H 0AP, UK; 2 School of Forestry & Environmental Studies, Yale UniversityNew Haven, CT 06511, USA

**Keywords:** Emotion, global warming, policy preferences

## Abstract

Prior research has found that affect and affective imagery strongly influence public support for global warming. This article extends this literature by exploring the separate influence of discrete emotions. Utilizing a nationally representative survey in the United States, this study found that discrete emotions were stronger predictors of global warming policy support than cultural worldviews, negative affect, image associations, or sociodemographic variables. In particular, worry, interest, and hope were strongly associated with increased policy support. The results contribute to experiential theories of risk information processing and suggest that discrete emotions play a significant role in public support for climate change policy. Implications for climate change communication are also discussed.

## 1. INTRODUCTION

Global warming is one of the world's most pressing problems. Unabated emissions of anthropogenic greenhouse gases, primarily from the burning of fossil fuels, are likely to have irreversible consequences.^1^ Substantial reductions in these emissions are therefore required if “dangerous” anthropogenic impacts are to be minimized, as recognized by international law.^2^ Alongside technological advances and coordinated international policies, the public will play an important role in global emissions reductions through energy use, consumer behavior, social norms, and, perhaps most importantly, support for climate and energy policies.

Americans have been somewhat concerned about global warming for many years,^3^ although in recent years, public concern about global warming has decreased.^4^–^6^ For example, in 2009 only 35% of Americans considered global warming a very serious problem compared to 44% in 2008.^4^ In a series of nationally representative surveys conducted between 2010 and 2012, Leiserowitz *et al*. found that fewer than 12% of Americans said they were “very worried” about global warming, an overall drop of 5 percentage points or more since 2008.^7^–^9^ A similar drop in public opinion has also been identified in comparable polls conducted internationally. Surveys conducted in the United Kingdom, for example, found that between 2005 and 2010, British public concern about the issue dropped approximately 10 percentage points.^10^–^12^ Several hypotheses have been proposed to explain this period of increased scepticism, including issue fatigue, the 2008 global financial crisis, and decreased media attention (see Pidgeon^13^ and Brulle *et al*.^14^ for reviews).

It is also important to situate these recent declines in historical context. A meta-analysis by Nisbet and Myers^3^ found that the proportion of Americans indicating global warming is personally important to them increased from 27% in 1997 to 52% in 2007. Public concern, however, did not rise steadily, with world events such as the 9/11 terrorist attacks temporarily lowering levels of concern. They also found that concern about global warming tends to be lower than concern for other environmental issues (e.g., water and air pollution).

As a national priority, climate change has always been lower than other economic and social issues. For example, in 2007, 68% of Americans said that the economy should be a top priority for the president and Congress compared to 38% who said global warming should be a top priority.^15^ Global warming's priority rating has also steadily declined over the past five years with only 26% in 2011 and 25% in 2012 saying global warming should be a top priority.^15^ Despite this, public support for a variety of national policies to reduce emissions remains high. For example, since 2010, approximately three-quarters of the American public has strongly or somewhat supported policies to fund more renewable energy research and regulate carbon dioxide as a pollutant.^16^

Researchers have investigated a range of factors that influence public responses to risks and hazards. The “risk as analysis” paradigm, for example, focuses on the use of cognitive deliberation to assess risk. Cognitive risk perception researchers have identified a variety of heuristics and biases used to process and understand risk information.^17^–^19^ More recent research, however, has focused on “risk as feelings,” arguing that people often rely more on affect and emotion than cognition when making risk judgments and decisions.^20^–^22^ Affect is processed quickly, automatically, and efficiently and enables people to make daily decisions with relatively little cognitive effort and studies have found that an “affect heuristic” is strongly associated with risk perceptions and policy support for a range of risk issues, including global warming.^23^–^26^

Slovic and Peters^21^ describe affect (feelings of good or bad) as a “faint whisper of emotion” (p. 322), but do discrete emotions also influence how people respond to global warming? If so, which emotions? Do they increase or decrease public support for climate-related policies? How well do discrete emotions predict public policy support compared to other known drivers such as affect, imagery, values, and demographic and political variables? Below, we review relevant research. To start, we consider how emotion is defined then review the existing research literature on the links between emotion and risk perception, positive emotions and attitudes, and emotions and policy support.

Researchers have investigated the content and function of emotion for many years. As such, the field has well-established definitions and conceptualizations of emotion.^27^–^29^ A comprehensive overview is beyond the scope of this article, but it is important to distinguish how discrete emotions differ from affect and what this distinction might offer to the study of how people process and make judgments about risk. Forgas^30^ defines emotions as “intense and short-lived” with a “definite cause and clear cognitive content” (p. 230). Affect, however, refers to a more general positive (good) or negative (bad) feeling.^31^ Emotions are often both more complex and less subtle. Anger and fear, for example, might evoke similar levels of negative affect, but are distinct emotions with separate causes, physiological expression, and cognitive content.^28^,^32^

Other research has examined the role of discrete emotions in risk perception. Sjoberg,^33^ for example, provided evidence that fear, anger, and worry influence public risk perceptions of mobile telephone use, genetically modified foods, and terrorism. Fear and anger have also been found to play an important role in risk perceptions of radiation sources,^34^ whereas disgust has been found to predict risk perceptions of food safety.^35^ Finucane^22^ argues that negative emotions are important determinants of risk perception because they motivate deeper information processing. Discussing mood, Schwarz *et al*.^36^ also argue that people process information more carefully and deliberately when negative moods are evoked. Using controlled experiments and drawing on models of persuasion (elaboration likelihood^37^ and heuristic systematic models^38^), Meijnders *et al*.^29^ examined the interactions between emotions and argument strength and found that greater fear of climate change was associated with greater systematic processing of information about energy-related behaviors.

Beyond the powerful influence of negative emotions, however, positive emotions can also have important effects. Sjoberg^33^ found that positive emotions, including interest, satisfaction, and optimism, were stronger positive predictors of attitudes toward nuclear waste repositories than negative emotions. Interest also accounted for a significant proportion of variance in attitude toward a variety of other risk issues, including “mad cow” disease, background radiation, and high-voltage power lines. Sjoberg argued that even though risks are perceived as threatening, people are often also motivated to feel hopeful and interested in options to mitigate the threat. In another study, Hoijer^39^ examined how the Swedish media communicated emotions in the social construction of global warming risk and found that hope and compassion were used as emotional anchors to help people understand projected climate impacts. These results suggest that many people do not view hazards merely as something to avoid. On the contrary, interest and hope may motivate people to learn more about the hazard and to take or support mitigation or adaptation measures.

There has been less research, however, on the influence of discrete emotions on policy support. A few researchers have explored the differential influence of fear and anger on policy preferences. For example, anger has been found to be strongly associated with support for vengeful or retribution-focused policy initiatives. Lerner *et al*.^32^ explored the effects fear and anger have on terrorism policy preferences and found that anger was more strongly associated with support for deportation policies than fear. Similarly, Nabi^40^ found that anger was more strongly associated with retributive drunk-driving policies. Even fewer studies, however, have explored the role of positive emotions on policy support. Truelove^41^ found that positive affect and discrete emotions were more strongly associated with support for wind energy than for coal or nuclear power. However, as far as we are aware, no studies have investigated the relationship of discrete emotions and global warming policy preferences. Rather than testing specific hypotheses, this exploratory study investigated whether different discrete emotions predict public support or opposition to global warming policies.

## 2. METHOD

### 2.1. Respondents and Procedure

A nationally representative survey of American global warming knowledge, risk perceptions, policy preferences, and behavior was conducted from late December 2009 to early January 2010, using the online, probability-based panel of Knowledge Networks.^3^ The survey had 1,001 adult respondents, with a 53% within-panel completion rate. The data were subsequently weighted to match U.S. Census Current Population Survey estimates of national demographic parameters, including gender, age, race, ethnicity, education, census region, and income. The margin of sampling error was plus or minus 3%, with 95% confidence.

### 2.2. Measures

#### 2.2.1. Policy Preferences

Respondents were asked to indicate their support for or opposition to a variety of different policies to mitigate global warming. Policies included research on renewable energy sources, the regulation of carbon dioxide as a pollutant, 25 cents per gallon increase in the gasoline tax, and establishment of a fund to make buildings more energy efficient. For analysis, a policy support index was created based on the overall mean response for each policy item (*α* = 0.90; see Table I for full questions).

**Table I:** Policy Support Index

			Alpha If Item	
	Mean	*SD*	Deleted	Alpha
Policy Support Index	2.61	0.70		0.90
Fund more research into renewable energy sources, such as solar and wind power	3.22	0.80	0.90	
Provide tax rebates for people who purchase energy-efficient vehicles or solar panels	3.07	0.84	0.89	
Regulate carbon dioxide	2.81	0.97	0.88	
Sign an international treaty to cut emissions	2.59	0.98	0.88	
Require electric utilities to produce at least 20% of their electricity renewables	2.55	1.01	0.88	
Cap and trade	2.46	0.89	0.89	
Establish a special fund to help make buildings more energy efficient	2.47	1.00	0.88	
Provide financial aid and technical support to developing countries that agree to limit their greenhouse gas emissions	2.37	0.97	0.88	
Increase taxes on gasoline	2.09	0.96	0.90	

*Note*: *n* = 974. Scales range from 1 (strongly oppose) to 4 (strongly support).

#### 2.2.2. Holistic Affect

Respondents were asked to rate whether global warming is a good or a bad thing using a unipolar, six-point Likert scale ranging between + 3 (very good) and –3 (very bad).

#### 2.2.3. Affective Imagery

Affective images^4^ were collected from all respondents and contain two elements: a cognitive component (the image category) and an associated affective rating (a goodness or badness evaluation). Images were collected using an open-ended word association methodology^44^,^45^ that enables context-free associations to emerge naturalistically. Images were collected by asking respondents to provide the first “word” or “phrase” that comes to mind when thinking about global warming. Responses took the form of single-word associations (e.g., “apocalypse”) or short narrative statements (e.g., “the end of the world”). Once collected, respondents were asked to provide an affective rating for the images they had provided using a six-point scale (where +3 = “a very good thing” and –3 = “a very bad thing”). This procedure produced a rich data set of images that were analyzed using a deductive content analysis procedure developed in earlier national studies.^(^46,24^)^ A total of 24 image categories were coded, but the top nine categories accounted for the majority of responses. These categories were not mutually exclusive, for example, a respondent associating global warming with “polar bears dying as sea ice melts” could be coded as both “nature” and “icemelt.” Ten percent of images were also double coded to ensure reliability of the coding frame and interreliability achieved satisfactory significance (80%). Discrepancies were resolved following discussion between coders. The mean affect of each image category was also calculated.

#### 2.2.4. Values

The cultural worldviews of egalitarianism and individualism were operationalized using a series of questions derived from cultural theory and from scales used by Dake,^47^,^48^ Peters and Slovic,^45^ Rippl,^49^ and Leiserowitz.^24^ For analysis, egalitarianism and individualism indices were created, each with a high reliability score (*α* = 0.78 and 0.85, respectively; see Table II for full questions).

**Table II:** Egalitarianism and Individualism Indices

	Mean	*SD*	Alpha If Item Deleted	Alpha
Egalitarianism Index (*n* = 928)	2.52	0.76		0.78
The world would be a more peaceful place if its wealth were divided more equally among nations.	2.29	0.99	0.73	
In my ideal society, all basic needs (food, housing, healthcare, education) would be guaranteed by the government for everyone.	2.33	1.06	0.71	
I support government programs to get rid of poverty.	2.73	0.92	0.73	
Discrimination against minorities is still a very serious problem in our society.	2.70	0.95	0.74	
Individualism Index (*n* = 934)	2.77	0.73		0.85
If the government spent less time trying to fix everyone's problems, we would all be a lot better off.	2.87	0.92	0.82	
Our government tries to do too many things for too many people. We should just let people take care of themselves.	2.66	0.94	0.81	
The government interferes too much in our everyday lives.	2.89	0.90	0.82	
Government regulation of business usually does more harm than good.	2.77	0.86	0.82	
People should be allowed to make as much money as they can, even if it means some make millions while others live in poverty.	2.66	0.95	0.85	

*Note*: Scales range from 1 (strongly disagree) to 4 (strongly agree).

#### 2.2.5. Emotions

Respondents were asked to rate the intensity of different emotions felt when thinking about global warming. The emotions assessed were derived from commonly used lists of primary and secondary discrete emotions 50 and included fear, helplessness, interest,^5^ anger, sadness, hope, depression, guilt, disgust, and worry. Respondents were asked: “How strongly do you feel each of the following emotions when you think about the issue of global warming?” Responses were recorded using a 1–4 scale, where 1 = not at all and 4 = very strongly.

#### 2.2.6. Sociodemographics

A range of sociodemographic information was also collected, including sex, age, race/ethnicity, educational attainment, political ideology (liberal–conservative), political party identification (Democrat, independent, Republican), religiosity (frequency of religious service attendance), and household income.

## 3. RESULTS

### 3.1. Policy Preferences

**Fig. 1:**
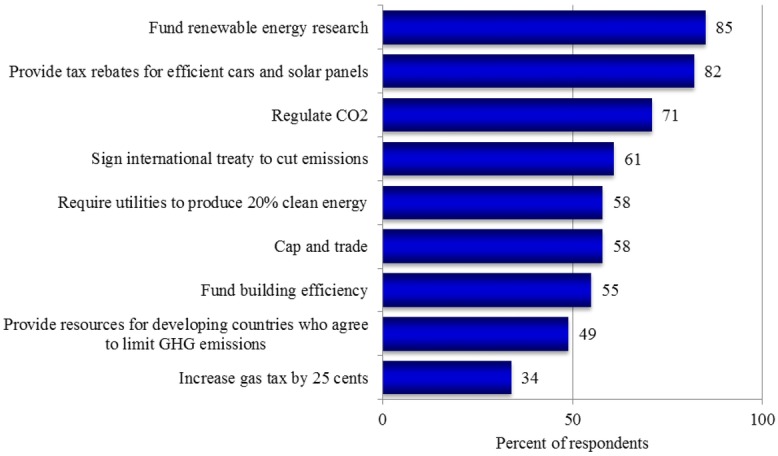
Support for policy option.

Respondents were asked how much they supported or opposed a range of different climate and energy-related policies (Fig. 1). Overall, respondents most strongly supported policies associated with renewable energy. Eighty-five percent supported funding more research into renewable energy sources including wind and solar power and 82% supported a policy to provide tax rebates for individuals who purchase energy-efficient vehicles or solar panels. Large majorities of respondents also supported the regulation of carbon dioxide as a pollutant (71%) and the signing of an international treaty to cut carbon dioxide emissions 90% by 2050 (61%). Fifty-eight percent supported cap and trade legislation to control the production of greenhouse gas emissions, but only when this policy was explained.^6^ A policy to increase gasoline taxes by 25 cents per gallon received the least public support (34%).

### 3.2. Emotional Responses to Global Warming

**Fig. 2:**
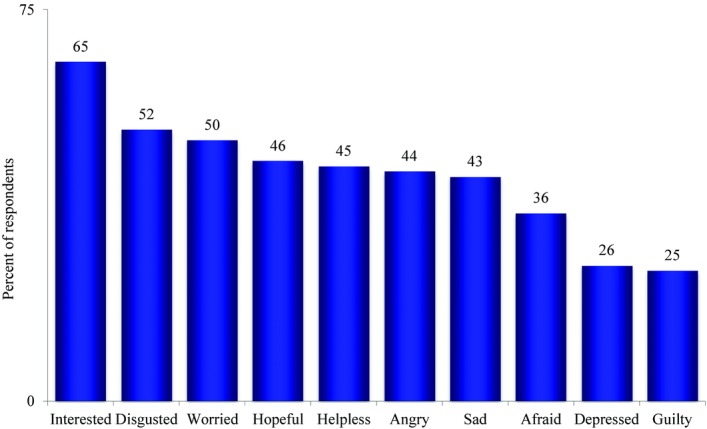
Emotions felt very or moderately in relation to global warming.

Respondents felt a variety of emotions when thinking about the issue of global warming (Fig. 2). Sixty-five percent said they felt moderately or very interested in global warming. Approximately half felt disgusted (52%), worried (50%), hopeful (46%), helpless (45%), angry (44%), or sad (43%) about the issue. Roughly, a third said they felt afraid (36%), whereas a quarter felt either depressed (26%) or guilty (25%).

### 3.3. The Influence of Emotions on Policy Support

Initially, a series of bivariate correlations were conducted to explore associations between the emotion variables and holistic affect (Table III). Some relatively high correlations were reported but multicollinearity statistics (tolerance and variance inflation factor (VIF)) were acceptable. A multiple regression model was then constructed, using the “enter” method, to explore the individual and combined association of holistic affect, affective imagery, values, discrete emotions, and sociodemographics variables with global warming policy preferences (Table IV). Only the items significant in separate linear regressions were entered into each model. The sample size of each regression analysis was also kept constant to enable comparison between models (*n* = 837). This sample size reflects the total number of participants for which there were no missing values in the full models of each regression analysis.

**Table III:** Correlation Matrix

	Holistic Affect	Worry	Afraid	Helpless	Interested	Angry	Sad	Hopeful	Depressed	Guilty
Worry	0.65**									
Afraid	0.43**	0.67**								
Helpless	0.37**	0.50**	0.64**							
Interested	0.32**	0.50**	0.57**	0.54**						
Angry	0.24**	0.41**	0.57**	0.48**	0.53**					
Sad	0.44**	0.59**	0.70**	0.58**	0.57**	0.68**				
Hopeful	0.24**	0.35**	0.36**	0.43**	0.64**	0.32**	0.38**			
Depressed	0.37**	0.55**	0.68**	0.57**	0.47**	0.57**	0.66**	0.27**		
Guilty	0.34**	0.54**	0.65**	0.51**	0.43**	0.41**	0.56**	0.35**	0.59**	
Disgusted	0.21**	0.40**	0.54**	0.52**	0.52**	0.78**	0.64**	0.26**	0.57**	0.40**

** Correlation is significant at the 0.01 level (two-tailed).

**Table IV:** Multiple Regressions on Policy Preferences

	Model 1	Model 2	Model 3	Model 4	Model 5	Model 6
Independent Variables	Affect	Images	Values	Emotions	Sociodemographics	Full
Holistic affect	0.53***					0.10**
Alarmists		0.03				–0.05*
Naysayers		–0.46***				–0.08**
Ozone		0.03				0.03
Do not know		–0.09**				–0.03
Icemelt		0.13***				0.07**
Pollution		0.05				0.01
Flood/sea level		0.09**				0.02
Politics		–0.18***				–0.06**
Dry/desert		0.05				0.02
Greenhouse		0.05				0.00
Egalitarianism			0.37***			0.18***
Individualism			–0.36***			–0.12***
Afraid				–0.03		–0.01
Helpless				–0.02		–0.05
Interested				0.12***		0.12**
Angry				–0.04		–0.01
Sad				0.04		–0.05
Hopeful				0.19***		0.16***
Depressed				0.06		0.07*
Guilty				0.10**		0.06*
Disgusted				–0.12**		–0.04
Worry				0.49***		0.25***
Worry*Hope				–0.03		0.00
Worry*Disgust				0.09**		0.07**
Party identification					0.18***	0.00
Political ideology					0.35***	0.05
Religiosity					0.00	0.01
Gender					0.05	0.00
Race/ethnicity					–0.06*	–0.04
*F*	330.60***	41.47***	240.83***	70.23***	50.31***	41.57***
Adjusted *R*^2^	0.28	0.33	0.37	0.50	0.23	0.59
*N*	837	837	837	837	837	837

*Note:* Dependent variable: Policy support index. Entries are standardized regression coefficients (betas).

* Significant at 0.05;

** significant at 0.01;

*** significant at 0.001.

#### 3.3.1. Global Warming Policy Support

*Model 1: Affect* found that holistic affect was a significant predictor and explained 28% of the variance in policy support (*F* (1, 835) = 330.60, *p* < 0.001, Adj. *R*^2^ = 0.28). The more negative respondents felt global warming to be the more likely they were to support a range of climate and energy-related policies. *Model 2: Images* found that several affective images were significantly associated with policy support or opposition and explained 33% of the variance (*F* (10, 826) = 41.47, *p* < 0.001, Adj. *R*^2^ = 0.33). Respondents, who provided naysayer, don't know, and politics-related images were more likely to oppose national policies, whereas those who provided icemelt and flooding/sea level rise images were more likely to support national policies. *Model 3: Values* found that egalitarian and individualistic values were significantly associated with policy support and opposition and explained 37% of the variance (*F* (2, 834) = 240.83, *p* < 0.001, Adj. *R*^2^ = 0.37). Egalitarians were more likely to support policies, whereas individualists were more likely to oppose them.

*Model 4: Emotions* explained 50% of the variance (*F* (12, 824) = 70.23, *p* < 0.001, Adj. *R*^2^ = 0.50). Worry about global warming was the strongest positive predictor of support for national policies, followed by hope and interest. Guilt was also weakly associated with policy support. Disgust was associated with opposition to climate and energy policies, likely reflecting the emotional response of the respondents most dismissive of the issue. The interaction between worry and disgust was also weakly positively associated with policy support, indicating that the respondents most worried *and* disgusted about global warming were more likely to support national policies.^7^ *Model 5: Sociodemographics* explained 23% of the variance (*F* (5, 831) = 50.31, *p* < 0.001, Adj. *R*^2^ = 0.23) in climate change policy support. Political ideology was the strongest predictor, with political liberals more likely to support climate policies and conservatives more likely to oppose them. Democrats were also more likely to support policies, whereas Republicans were not. Finally, nonwhite Americans were slightly more likely to support global warming policies.

In *Model 6: Full* all variables were entered to identify the strongest predictors of climate change policy support and opposition. The full model explained 59% of the variance (*F* (30, 806) = 41.57, *p* < 0.001, Adj. *R*^2^ = 0.59) in policy support. Worry was the strongest predictor followed by egalitarianism, that is, respondents who worried about global warming or who held an egalitarian worldview were more likely to support climate policies. Interest, hope, and negative holistic affect were also each strongly associated with support for national policies. Individualism was the strongest predictor of policy opposition, followed by naysayer and politics imagery. That is, respondents who held an individualistic worldview and who provided skeptical and political associations to global warming were more likely to oppose national policies.

## 4. DISCUSSION

This investigation explores the role discrete emotions play in public climate change policy preferences. Previous research has documented the important role of affect as a subtle form of emotion,^(^23,46,24,52^)^ but fewer studies have explored the role discrete emotions play in policy support. This research found that discrete emotions alone were able to explain a large proportion of the variance (50%) in public global warming policy support. Further, discrete emotions were the strongest predictors of policy support, even controlling for other factors like holistic affect, imagery, values, sociodemographics, political party, and ideology.

Worry, in particular, was the single strongest predictor. That is, the more respondents worried about global warming, the more likely they were to support national climate and energy policies. Interestingly, however, fear was not associated with increased policy support in either the emotion block or the full model. Although a positive correlation was found between worry and fear in initial bivariate correlation analyses, the relative impact of fear was “washed out” when combined with other items in both of these models. This finding has important implications for climate change educators and communicators. Fear appeals have often been used under the assumption that scaring the public about climate change will engage them in the issue, motivate individual action, and generate public support for broad policy change, but recent research demonstrates that fear appeals are often ineffective or even counterproductive. “Dire” fear-based messaging around extreme weather and other climate phenomena^(^39,53^)^ has been found to raise anxieties, but also to distance the public.^54^ O'Neill and Nicholson-Cole^55^ found that catastrophic and alarmist visual imagery actually decreased public engagement with the issue. When frightened about a threat that seems individually uncontrollable, many individuals purposively disengage, via psychological distancing, as a form of emotion-focused coping.^56^ Fear appeals have also been tested by health communication researchers, who have also found that they can be counterproductive, especially in the absence of messages that increase perceived self-efficacy.^57^

Moser^58^ argues that fear can cause attitude and behavioral change but only in situations where the individuals feel personally “at risk,” among other factors. The limited success of global warming fear appeals may also be attributable to a feeling of personal invulnerability combined with the belief that individual or collective action either is too difficult or would not make a difference. As many Americans view climate change as a relatively abstract and distant threat,^46^,^59^ the challenge for climate communicators is to increase both the sense of threat while also increasing the sense of personal and collective efficacy.

By contrast, worry was the strongest predictor of public support for global warming policies, suggesting that perhaps “worry appeals” should be a focus for risk communicators. “Worry appeals” might promote a more sustainable and constructive emotional engagement with the issue of global warming. By contrast, fear is an intense emotion typically experienced in response to a perceived immediate threat and primes the body for immediate action, including the fight or flight reflex.^60^ Similarly, intense fear can cause an “amygdala hijack,” reducing cognitive and analytical processing of risk information.^61^ Climate change, however, is a long-term incremental threat that will manifest over decades and a prototypical example of a “hidden hazard”—“risks that despite potentially serious consequences for society, generally pass unheeded until they reach disaster proportions,”^62^ p. 251.^62^,^63^ In fact, this and prior studies have documented that climate change is often perceived as a threat distant in time and space by many members of the public,^46^,^64^ exactly the kind of hazard that is less likely to activate a full-blown fear response. In addition, fear-based communications tend to emphasize apocalyptic, worst-case scenarios, which in turn can cause issue avoidance or even hostile backlash among some audiences, leading them to disengage, doubt, or dismiss the issue.^65^,^66^

We suggest, however, that worry is a less intense emotion better suited to the issue of climate change. Worry tends to motivate, not short-circuit, more intense cognitive and analytical processing of risk information. People often worry about their careers, health, retirement, the state of the economy, or their children's future, leading them to seek out additional information about the risks as well as potential actions to reduce risk. Higher levels of worry about cancer, for example, have been found to predict greater attention to cancer-based health information^67^ and interest in genetic testing and beliefs about its benefit.^68^ Although people appear to have a “finite pool of worry,”^69^ “worry appeals” could prove more effective than “fear appeals” as a means to motivate constructive engagement with climate change. Worry can motivate and promote processes of problem identification, analysis, option seeking, deliberative decision making, implementation, evaluation, and recalibration—in short, the kind of deliberative and iterative decision making climate change requires.^62^ Worry can also, under certain circumstances, be useful for developing strategies to cope with or have control over stressful events, particularly in situations with a high degree of perceived personal investment.^70^ Extreme levels of worry, however, can become debilitating,^71^,^72^ so “worry appeals” would still need to be carefully calibrated to be neither too mild nor too intense.

This study also found that positive emotions appear to play an important role in public support for climate policies. Interest and hope were strongly associated with greater policy support. Similarly, Sjoberg^33^ reported strong associations between interest and demand for risk mitigation across a variety of hazards, and Simons *et al*.^73^ found that hope played a key role in public responses to nanotechnology, including expectations about its potential benefits. Meneses^74^ argues that campaigns should use positive rather than negative rhetoric to promote recycling behavior, as the act of recycling and other pro-environmental behavior is often associated with positive emotions. Feeling good about doing the “right thing” can be an important motivator of behavior change.^75^,^76^ Arguably, interest increases issue salience, information seeking, and learning, whereas the lack of interest leads to public disengagement or apathy. Similarly, hope aligned with personal or collective efficacy supports individual and collective action.^77^

In an experimental study, Myers *et al*.^78^ presented subjects with climate change information using one of three frames (health, national security, and environmental) and assessed their hope versus anger-based emotional reactions. They found that presenting global warming as a health issue was more likely to promote feelings of hope than either the national security or environmental frames. Promoting feelings of hope about mitigation policies that also benefit human health could be an effective means of communicating climate change to both engaged and disengaged audiences. Such a “gain frame advantage”^79^ has been found to promote more positive attitudes toward climate change mitigation than information focused on losses, or costs of inaction.

Elaboration likelihood models of persuasion^37^ also suggest that positive rather than negative emotions are more persuasive and likely to sustain enduring attitudes over time for issues of low involvement, that is, for issues where people do not see themselves personally “at risk” or vulnerable.^80^ Given the general lack of public involvement with the issue of climate change, combined with the relationship between hope, interest, and policy support found in this investigation, developing communications that increase public interest, inspire hope, and encourage positive feelings when people act in climate-friendly ways may be more effective than fear or guilt appeals. This study also found that many Americans are interested in the issue and hopeful about policies to mitigate the risk. As a consequence, climate change communicators should also consider using “interest appeals” and “hope appeals” to promote constructive engagement with climate change solutions.

Finally, this study also found that discrete emotions explained more variance than either negative affect or cultural worldviews. Although previous research has documented the important role of affect and worldviews in global warming policy support,^24^ discrete emotions were the most powerful variables in this study. These findings thus support the “risk as feelings” hypothesis that experiential factors, including affect and discrete emotions, play a critical role in the processing of risk information.^20^,^26^

As an exploratory study, a limitation is the correlational nature of the research findings. Due to the cross-sectional nature of the survey data, we cannot determine the causal relationship between variables, although it is unlikely, for example, that greater support for climate policies leads to increased levels of worry about climate change. This study also did not assess trait emotions, that is, dispositional emotions such as the tendency for some people to worry, be fearful, or be hopeful more than others, as opposed to specific levels of worry, fear, or hope about climate change. The emotion measures in this study were also based on respondents’ self-reported assessments of their emotional reactions, not direct physiological measures. Further research will be required to determine the specific relationships between each emotion and policy support. Further research will also be required to more fully understand different emotional responses to different aspects of global warming. Each of the causes, consequences, and solutions to climate change may evoke different emotional responses among different people.^23^ For example, windmills clearly activate different emotions and interpretations in different members of the public. The relative influence of positive versus negative emotions on public responses to global warming will be especially important to explore in this regard. For example, it will be interesting for further research to investigate when and to what extent positive emotions associated with potential solutions to global warming might matter more than negative emotions associated with the impacts. The present analysis, however, is an initial exploration of the variety of discrete emotions Americans associate with the issue of global warming in general—an important level of analysis in its own right as “global warming,” as a holistic term, is frequently used in public and policy discourse.

In summary, this research found that discrete emotions—especially worry, interest, and hope—appear to have a large influence on American climate change policy preferences. The challenge for communication strategists is how best to cue these powerful motivations to promote public engagement with climate change solutions.

## Footnotes

3 Knowledge Networks provides panel members without a computer or Internet connection each of these services.

4 “Imagery” refers to mental representations or cognitive content within the individual mind and can include both perceptual and symbolic representations.^42^ “Affective imagery” is therefore defined as “sights, sounds, smells, ideas, and words, to which positive and negative affect or feeling states have become attached through learning and experience” (p. 3).^43^

5 Interest has been categorized as an “eccentric” emotion but one that has a stable pattern of underlying cognitive appraisal.^51^

6 A definition of cap and trade was provided to all respondents before they were asked about their support or opposition for the policy.

7 Exploratory tree analysis in SPSS identified several interactions to explore: worry × disgust, worry × afraid, worry × interest, worry × guilt, and worry × hope. However, separate linear regressions with policy support identified that only worry × disgust and worry × hope were significant and therefore only these were included in the multiple regressions.
